# Plasma levels of advanced glycation endproducts are associated with type 1 diabetes and coronary artery calcification

**DOI:** 10.1186/1475-2840-12-149

**Published:** 2013-10-17

**Authors:** Marcelle GA van Eupen, Miranda T Schram, Helen M Colhoun, Jean LJM Scheijen, Coen DA Stehouwer, Casper G Schalkwijk

**Affiliations:** 1 Department of Internal Medicine, Maastricht University Medical Centre (MUMC) and Cardiovascular Research Institute Maastricht (CARIM), Universiteitssingel 50, Maastricht 6200, MD, the Netherlands; 2 Biomedical Research Institute, University of Dundee, Dundee, Scotland

**Keywords:** Advanced glycation endproducts, Coronary artery calcification, Type 1 diabetes

## Abstract

**Background:**

Advanced glycation endproducts (AGEs) may play a role in the development of coronary artery calcification (CAC) in type 1 diabetes (T1DM). We studied plasma AGEs in association with T1DM and CAC, and whether or not the latter association could be explained by low-grade inflammation (LGI) or endothelial dysfunction (ED).

**Methods:**

We studied 165 individuals with and 169 without T1DM. CAC was quantified in a CAC score based on CT-scanning. Plasma levels of protein-bound pentosidine, N^ϵ^-(carboxymethyl)lysine (CML) and N^ϵ^-(carboxyethyl)lysine (CEL) were measured with HPLC/UPLC with fluorescence detection or tandem-mass spectrometry. Tetrahydropyrimidine (THP) was measured with ELISA, as were HsCRP, and sVCAM-1 and vWF, as markers for LGI and ED, respectively. Associations were analyzed with ANCOVA and adjusted for age, sex, BMI, waist-to-hip ratio, smoking, blood pressure, lipid profile, eGFR and T1DM.

**Results:**

Individuals with T1DM had higher plasma levels of pentosidine, CML and THP compared with controls; means (95% CI) were 0.69 (0.65-0.73) vs. 0.51 (0.48-0.54) nmol/mmol LYS, p < 0.001; 105 (102–107) vs. 93 (90–95) nmol/mmol LYS, p < 0.001; and 126 (118–134) vs. 113 (106–120) U/mL, p = 0.03, respectively. Levels of pentosidine were higher in individuals with T1DM with a moderate to high compared with a low CAC score, means (95% CI) were 0.81 (0.70-0.93) vs. 0.67 (0.63-0.71) nmol/mmol LYS, p = 0.03, respectively. This difference was not attenuated by adjustment for LGI or ED.

**Conclusions:**

We found a positive association between pentosidine and CAC in T1DM. These results may indicate that AGEs are possibly involved in the development of CAC in individuals with T1DM.

## Background

Individuals with type 1 diabetes mellitus (T1DM) have an increased risk of cardiovascular disease (CVD) [1]. Advanced glycation endproducts (AGEs), which are formed by a nonenzymatic reaction between reducing sugars and proteins, are thought to play an important role in the development of CVD in T1DM [2,3]. AGEs are able to affect cell function via intracellular glycation of proteins, altering their function [2], via cross-linking of extracellular matrix proteins in large arteries, resulting in arterial stiffness [4], and by binding to the receptor for AGEs (RAGE), inducing receptor-mediated cell activation [5,6]. Well-studied AGEs are the cross-link AGE, pentosidine, and Nϵ-(carboxymethyl)lysine (CML), i.e. a ligand for RAGE. More recently, methylglyoxal (MGO) has gained increased attention as being the most important precursor of rapid intracellular production of AGEs [2,7]. MGO primarily reacts with arginine to form 5-hydro-5-methylimidazolone (MG-H1), tetrahydropyrimidine (THP) and argpyrimidine, and with lysine to form N^ϵ^ (carboxyethyl)lysine (CEL) and methylglyoxal lysine dimer (MOLD) [8,9].

Surprisingly, large studies comparing plasma AGE levels between individuals with and without T1DM are scarce. So far, most of them are based on relatively small patient and control groups [10,11], have used non-specific immunological techniques for the detection of AGEs [12,13] or concentrate on individuals with T1DM only. In individuals with T1DM, we recently demonstrated that plasma AGEs are positively associated with incident fatal and nonfatal CVD, independent of cardiovascular risk factors [3].

Coronary artery calcification (CAC) is a well-accepted early marker and precursor of CVD [14,15] and correlated with total atheroma burden. Individuals with T1DM have been shown to have more coronary CAC [16,17]. In individuals with kidney disease, the AGE pentosidine and the AGE-RAGE axis have been associated with arterial calcification [14,18]. In addition, experimental studies recently demonstrated that AGEs are able to induce vascular calcification [19-23]. However, no study has previously investigated the association of plasma AGEs with CAC in individuals with T1DM. We hypothesised that AGEs could lead to CVD either by increasing CAC directly or more indirectly by increasing the atherosclerotic process in individuals with T1DM. Potential mechanisms through which AGEs could lead to the development of CAC are via low-grade inflammation (LGI) and endothelial dysfunction (ED), which are both associated with higher AGE levels [3].

In view of the above, the aims of this study were to examine, first, whether plasma levels of the AGEs pentosidine, CML, CEL and THP are higher in a large sample of individuals with T1DM, compared to controls; second, if these AGEs are associated with CAC in individuals with T1DM; and third, to which extent the association of AGEs with CAC could be explained (i.e. mediated) by markers of LGI and ED.

## Methods

### Study population and design

In 1998, a random sample of 199 men and women with T1DM aged 30 to 55 years was taken from the diabetes registers of five London hospitals. T1DM was defined by age of onset ≤ 25 years and insulin treatment within one year of diagnosis. A random sample of 201 individuals from the general population, stratified to have a similar age and gender distribution to the group with diabetes, was drawn from the lists of two London general practices. It was confirmed that these individuals had no clinical history of diabetes and were not on any treatment for diabetes. Participants were included regardless of any history of heart disease. One participant (a woman with diabetes) had a history of angina; none had had a myocardial infarction. Pregnant women and patients on renal replacement therapy were excluded. Ethics Committee approval was obtained. All participants gave informed written consent prior to participation, having received full details of the study procedures. Further details of this study have been described elsewhere [16]. Individuals with missing data on plasma AGEs (n = 48) or on any of the potential confounders (n = 18), i.e. on triglycerides (n = 13), HDL (n = 1), LDL (n = 18) or eGFR (n = 1), were excluded from our analyses. Individuals with incomplete data did not materially differ in baseline characteristics from individuals with complete data, except for the percentage of individuals with a CAC score >10. We excluded 22 individuals with a CAC score >10 because of missing data on AGEs (n = 20), or potential confounders (n = 2). The complete case analysis included 165 individuals with and 169 without T1DM (n = 334).

### Analysis of protein bound AGEs and lysine in plasma

Plasma AGEs were measured in EDTA samples obtained from fasting venous blood, which were stored at -80°C until analysis. Protein-bound pentosidine was quantified using HPLC with fluorescence detection, as described in detail elsewhere [24]. Intra- and interassay coefficients of variation (CVs), as analysed in this study, for pentosidine were 3.8 and 6.9%, respectively. Protein-bound CML, CEL and lysine were quantified using UPLC MS/MS [25]. Intra- and interassay CVs were 4.5 and 3.4% for CML, 5.4 and 18.1% for CEL and 5.0 and 5.0% for lysine, respectively. THP was measured using competitive ELISA [26]. Intra- and interassay variations were 5% and 8%, respectively. Concentrations of protein-bound pentosidine, CML and CEL were adjusted for levels of lysine and expressed as nmol/mmol lysine.

### EBCT scan

An Ultrafast CT scanner (IMATRON C-150XL) was used to quantify coronary calcification. Two sets of 20 transverse tomograms of 3-mm thickness were obtained from the lower margin of the bifurcation of the right branch of the pulmonary artery to the apex of the heart with breath holding of the individual. A radiologist placed a region of interest around each potentially calcific lesion (peak density <130 Hounsfield U) within the right coronary, circumflex, left anterior descending and left main coronary arteries. The area and peak density of each lesion was measured. A density score of 1 to 4 was defined based on the peak density of the lesion; the calcification score was then calculated as the product of the area of the lesion and its density score as described by Agatston et al. [27]. To be included in the calcification score a lesion had to have an area of at least 0.51 mm2, i.e., two contiguous pixels and a peak density of at least 130 Hounsfield U. A total score for each artery and for the entire heart was calculated by summing the lesion scores. The radiation exposure was <1 mSv. All scans were scored by the same radiologist, who was blinded to the gender and the diabetes status of the individual. Based on a small repeatability study (n = 20) the within-observer agreement for the presence of any calcification was high (kappa = 0.84).

### Coronary artery calcification score

The cut-off value of 10 for the CAC score was based on a recent review and meta-analysis on the validation of electron beam computed tomography for coronary artery disease in both symptomatic and asymptomatic individuals [28]. In this review, the CAC score was divided in three groups: low (0–10), moderate (10–400) and high (>400). Since only 3 individuals had a CAC score above 400, the CAC score was analysed dichotomously comparing individuals with a CAC score of 0–10 (low) to individuals with a CAC score of >10 (moderate to high).

### Covariates

Glomerular filtration rate (eGFR) was estimated according to the short Modification of Diet in Renal Disease equation (MDRD) = 186* [serum creatinine (mg/dL)]^-1.154^* [age (y)]^-0.203^* [0.742 if patient is female] [29]. Measurement of creatinine was based on the enzymatic method. HbA1c, systolic and diastolic blood pressure, total cholesterol, HDL and LDL cholesterol and triglycerides were measured as described elsewhere [16]. High-sensitivity C-reactive protein (hsCRP), a marker of LGI, was measured with a highly sensitive in-house ELISA, as described previously [30]. A commercially available ELISA kit was used to measure plasma soluble vascular cell adhesion molecule 1 (sVCAM-1) (R&D Systems). Von Willebrand factor (vWF) activity was measured in heparin plasma by Shield vWF activity ELISA kit (Shield Diagnostics Ltd, Dundee, Scotland) using IgG monoclonal antibodies, and expressed as percentage of vWF in pooled plasma of healthy volunteers. sVCAM-1 and vWF were considered markers of ED.

### Statistical methods

Analyses were carried out using SPSS version 20 for Windows. Comparisons of baseline characteristics between groups were made by use of a Student’s *t* or χ^2^ tests. In Table 1, in case of CAC score levels, comparisons were made by use of a Mann–Whitney U test. We performed complete case analyses. ANCOVA was used to identify the differences in levels of plasma AGEs between individuals with and without T1DM and with and without a CAC score >10. Variables with a skewed distribution (e.g. plasma pentosidine and THP) were log_10_-transformed, and transformed back to provide geometric means. We investigated whether the associations of AGEs with CAC differed between men and women by adding interaction terms to the models. Finally, linear regression analysis was used to evaluate to what extent LGI and ED mediated the association of AGEs with CAC in individuals with T1DM. The percentage change in the magnitude of the linear regression coefficient between the model with or without LGI and/or ED reflects to what extent the association could be explained by mediation. P-values <0.05 were considered statistically significant, except for interaction terms, where a p-value <0.10 was considered statistically significant.

**Table 1:** General characteristics of the coronary artery calcification study (CACS)

	**Controls (n = 169)**	**T1DM (n = 165)**	**p-value**
*General*			
Age (years)	38.0 ± 3.8	37.8 ± 4.3	0.75
Sex (number of males/females)	72/97	83/82	0.16
Diabetes duration (y)	-	23.5 ± 7.6	-
HbA1c (%)	5.31 ± 0.42	8.83 ± 1.53	<0.001
Smoking, former or current (n (%))	84 (50)	75 (46)	0.44
Pack-years of smoking for former or current smokers (y)	8.6 [3.5-17.0]	10.0 [4.5-18.0]	0.87
BMI (kg/m^2^)	25.2 ± 4.5	25.4 ± 3.6	0.53
Waist-to-hip ratio	0.85 ± 0.08	0.87 ± 0.08	0.14
Total cholesterol (mmol/L)	5.35 ± 1.02	5.32 ± 1.06	0.75
HDL cholesterol (mmol/L)	1.73 ± 0.40	1.84 ± 0.47	0.02
LDL cholesterol (mmol/L)	3.07 ± 0.93	2.95 ± 0.93	0.24
Triglycerides (mmol/L)	1.06 [0.76-1.44]	0.97 [0.77-1.35]	0.37
Systolic BP (mmHg)	117 ± 14	124 ± 14	<0.001
Diastolic BP (mmHg)	72 ± 10	73 ± 9	0.22
eGFR_MDRD_ (ml/min/1.73 m^2^)	89 ± 16	97 ± 15	<0.001
CAC score (units)	0.0 [0.0-1.0]	1.0 [0.0-5.0]	<0.001
CAC score > 10 (n (%))	2 (1)	30 (18)	<0.001
hsCRP (mg/L)	0.84 [0.43-1.76]	1.04 [0.46-2.87]	0.02
sVCAM-1 (ng/mL)	985 [820–1175]	1161 [964–1374]	<0.001
vWF (%)	87 [65–108]	97 [72–126]	<0.01
*Advanced glycation endproducts*			
Pentosidine (nmol/mmol LYS)	0.48 [0.41-0.58]	0.65 [0.55-0.81]	<0.001
CML (nmol/mmol LYS)	92.5 ± 15.7	104.6 ± 19.4	<0.001
CEL (nmol/mmol LYS)	14.5 ± 3.2	15.1 ± 3.7	0.12
THP (U/mL)	110.0 [94.0-123.2]	117.8 [102.9-135.4]	0.02

Data are presented as mean ± standard deviation (SD), in case of a normal distribution of data, or as median [inter quartile range (IQR)], in case of a skewed distribution of data, unless otherwise indicated.

HbA1c, glycated hemoglobin; BMI, body mass index; HDL, high-density lipoprotein; LDL, low-density lipoprotein; BP, blood pressure; eGFR_MDRD_, estimated Glomerular Filtration Rate by abbreviated Modification of Diet in Renal Disease equation; CAC, coronary artery calcification; hsCRP, high sensitivity C-reactive protein; sVCAM-1, soluble vascular cell adhesion molecule 1; vWF, von Willebrand Factor; CML, N(epsilon)-(carboxymethyl)lysine; CEL, N(epsilon)-(carboxyethyl)lysine; THP, tetrahydropyrimidine.

## Results

Table 1 shows the general characteristics of the study population. HbA1c levels were higher in individuals with T1DM, as were HDL cholesterol, systolic blood pressure (SBP), eGFR, hsCRP, sVCAM-1 and vWF. The percentage of individuals with a CAC score >10, compared to 0–10, was higher in T1DM than in controls; only two individuals without T1DM had a CAC score >10, whereas 32 individuals with T1DM had a CAC score >10 (Table 1).

### Plasma AGEs in individuals with and without T1DM

Plasma levels of the AGEs pentosidine, CML and THP were significantly higher in individuals with T1DM compared to controls (Table 1). Plasma levels of pentosidine, CML and THP remained significantly higher in individuals with T1DM after adjustment for age and sex and additional adjustment for systolic and diastolic blood pressure, body mass index (BMI), waist to hip ratio (WHR), smoking, LDL and HDL cholesterol, triglycerides and eGFR (Table 2). In individuals with T1DM, none of the plasma AGEs were significantly associated with HbA1c or diabetes duration (data not shown).

**Table 2:** Plasma AGE-levels in individuals with and without T1DM

		**Model 1**			**Model 2**		
		**Mean**	**95%-CI**	**p-value**	**Mean**	**95%-CI**	**p-value**
Pentosidine* (nmol/mmol LYS)	*control*	0.51	0.48 – 0.53		0.51	0.48 – 0.54	
	*T1DM*	0.69	0.65 – 0.73	<0.001	0.69	0.65 – 0.73	<0.001
**CML** (nmol/mmol LYS)	*control*	92.3	89.6 – 94.9		92.6	90.1 – 95.1	
	*T1DM*	104.8	102.1 – 107.5	<0.001	104.5	102.0 – 107.0	<0.001
**CEL** (nmol/mmol LYS)	*control*	14.5	14.0 – 15.0		14.6	14.1 – 15.2	
	*T1DM*	15.0	14.5 – 15.5	0.212	14.9	14.4 – 15.4	0.489
THP* (U/mL)	*control*	112.7	106.2 – 119.7		112.7	105.7 – 119.9	
	*T1DM*	125.6	118.0 – 133.4	0.015	125.6	117.8 – 134.0	0.025

Data are presented as adjusted means or geometric means* of AGEs in individuals with and without T1DM, by use of a complete case analysis.

n = 169 for individuals without and n = 165 for individuals with T1DM.

Model 1 was adjusted for age and sex. Model 2 was adjusted for age, sex, SBP, DBP, BMI, WHR, pack-years of smoking, LDL, HDL, triglycerides and eGFR.

CML, N(epsilon)-(carboxymethyl)lysine; CEL, N(epsilon)-(carboxyethyl)lysine; THP, tetrahydropyrimidine.

### Plasma AGEs in individuals with T1DM and a low or moderate to high coronary artery calcification score

The associations of AGEs with CAC were analysed in T1DM only, due to the low number of individuals with a CAC score >10 in the control group. We performed a complete case analysis in the 165 individuals with T1DM, of whom 135 had a CAC score of 0–10 and 30 had a score >10. The associations of AGEs with the CAC score did not significantly differ between men and women; therefore we presented the results for men and women combined. Plasma levels of the AGE pentosidine were significantly higher in individuals with a CAC score >10 compared to those with a score of 0–10 after adjustment for age and sex, while levels of CML, CEL and THP were similar (Table 3). After additional adjustment for systolic and diastolic blood pressure, BMI, WHR, smoking, LDL and HDL cholesterol, triglycerides and eGFR, plasma pentosidine levels remained significantly higher in individuals with a CAC score >10 (Table 3). Additional adjustment for HbA1c level and diabetes duration did not materially change these results. The association of plasma pentosidine with the CAC score was not explained (i.e. mediated) by LGI or ED (Table 4).

**Table 3:** Plasma AGE-levels in individuals with T1DM and a moderate to high compared with a low CAC score

		**Model 1**			**Model 2**		
		**Mean**	**95%-CI**	**p-value**	**Mean**	**95%-CI**	**p-value**
Pentosidine* (nmol/mmol LYS)	*CAC = 0-10*	0.67	0.63 – 0.72		0.67	0.63 – 0.71	
	*CAC > 10*	0.80	0.69 – 0.92	0.034	0.81	0.70 – 0.93	0.028
**CML** (nmol/mmol LYS)	*CAC = 0-10*	105.0	101.7 – 108.2		104.5	101.5 – 107.5	
	*CAC > 10*	102.9	95.8 – 110.0	0.608	105.0	98.3 – 111.8	0.883
**CEL** (nmol/mmol LYS)	*CAC = 0-10*	14.9	14.2 – 15.5		14.9	14.3 – 15.5	
	*CAC > 10*	15.9	14.6 – 17.3	0.166	15.9	14.6 – 17.3	0.163
THP* (U/mL)	*CAC = 0-10*	122.2	113.5 – 131.8		121.6	112.7 – 131.2	
	*CAC > 10*	140.6	119.4 – 165.6	0.131	143.9	121.3 – 170.6	0.086

Data are presented as adjusted means or geometric means* of AGEs in individuals with a CAC score of 0–10 compared to >10 in individuals with T1DM, by use of a complete case analysis.

n = 135 for individuals with a calcification score of 0–10; n = 30 for individuals with a calcification score of >10.

Model 1 was adjusted for age and sex. Model 2 was adjusted for age, sex, SBP, DBP, BMI, WHR, pack-years of smoking, LDL, HDL, triglycerides and eGFR. CML, N(epsilon)-(carboxymethyl)lysine; CEL, N(epsilon)-(carboxyethyl)lysine; THP, tetrahydropyrimidine.

**Table 4:** Mediation analyses of the association of plasma pentosidine levels with the CAC score by low grade inflammation and endothelial dysfunction in T1DM

	**Model 2**			**Model 2 + LGI**			**Model 2 + ED**		
	**Standardised β**	**95%-CI**	**p-value**	**Standardised β**	**95%-CI**	**p-value**	**Standardised β**	**95%-CI**	**p-value**
**Pentosidine** (SD)	0.47	0.03 – 0.91	0.036	0.47	0.03 – 0.91	0.038	0.43	-0.02 – 0.87	0.060

Data are presented as standardized β (sβ). A sβ of 0.47 SD indicates that individuals with T1DM with a CAC score of >10 have on average 0.47 SD higher pentosidine levels compared to individuals with a score of 0–10.

n = 122 for individuals with a calcification score of 0–10; n = 27 for those with a calcification score of >10.

Model 2 was adjusted for age, sex, SBP, DBP, BMI, WHR, pack-years of smoking, LDL, HDL, triglycerides and eGFR. This model was additionally adjusted for high sensitivity C-reactive protein (hsCRP), a marker for low-grade inflammation (LGI), or for soluble vascular cell adhesion molecule 1 (sVCAM-1) and von Willebrand Factor (vWF), which are markers of endothelial dysfunction (ED).

## Discussion

Our study had three main findings. First, we found higher plasma levels of the AGEs pentosidine, CML and THP in individuals with T1DM. Second, the AGE pentosidine was positively associated with CAC, an early marker of CVD. Third, the association of pentosidine with CAC was not explained by markers of LGI or ED.

### Plasma AGEs in type 1 diabetes

This is the first larger study that has quantified multiple plasma AGEs with state-of-the-art ultra-performance liquid chromatography (UPLC) in combination with tandem mass spectrometry or, in case of pentosidine, with high-performance liquid chromatography (HPLC) and fluorescence detection. These techniques are considered to be the most accurate techniques for the measurement of AGEs at this moment. Unfortunately, because of acid instability of THP, it was not possible to measure THP with these techniques and therefore THP was measured with an ELISA. We investigated four out of many different AGEs and found that plasma levels of the AGEs pentosidine, CML and THP were significantly higher in individuals with T1DM as compared to controls, independent of age, sex, systolic and diastolic blood pressure, BMI, WHR, smoking, LDL and HDL cholesterol, triglycerides and eGFR. Levels of CEL were not statistically different. These results are in agreement with previous studies which were confined to small study populations [10,11] and/or have used non-specific immunological techniques for the detection of AGEs [12,13].

We did not find any association of plasma AGEs with HbA1c in the individuals with T1DM. These findings are consistent with many other studies that reported no association of plasma AGEs with HbA1c [31-35]. An explanation for this lack of association could be that AGEs can also be formed through other pathways, for example lipid peroxidation, besides glucose metabolism. Moreover, HbA1c and AGEs presumably reflect different pathways following hyperglycaemia and different timeframes of hyperglycaemia.

### Associations between plasma AGEs and CAC in type 1 diabetes

We and others have previously shown that the prevalence of CAC is increased in T1DM [16,17]. In individuals with kidney disease, the AGE pentosidine and the AGE-RAGE axis have been associated with arterial calcification [14,18]. This is the first study that has examined the association of plasma AGEs with CAC in T1DM. We found higher levels of the plasma AGE pentosidine, but not CML, CEL and THP, in individuals with T1DM with a moderate to high compared to a low CAC score. These results are in line with a previous study that reported an independent positive association of pentosidine with CAC in individuals undergoing hemodialysis [14], while no association was found between CML and CAC. Moreover, Conway et al. and Orchard et al. showed that skin autofluorescence, as a possible reflectance of tissue AGE accumulation, was associated with CAC severity in T1DM [36,37].

### Potential mechanisms underlying the associations between AGEs and CAC

In contrast to the positive association of pentosidine with CAC, CML, CEL and THP were not associated with CAC. This could indicate that plasma pentosidine is a better reflection of total AGE formation than these other AGEs or that it is more precisely quantified with current methods. Alternatively, pentosidine differs from the other AGEs measured because it is known as a cross-linking AGE [4,38], and it might be that cross-linking AGEs in particular are linked to CAC. THP was borderline significantly associated with CAC (p = 0.09). THP is one of the AGEs, next to CEL, which is formed from the reaction of the reactive dicarbonyl MGO with arginine or lysine, respectively, predominantly formed from intracellular glycation [2]. Little is known about THP, but it could represent a better reflection of intracellular MGO-AGEs than CEL. Moreover, auto-antibodies against MGO-modified apolipoprotein B100 have been found to be inversely associated with CAC in patients with type 2 diabetes [39], also indicating a role of MGO in CAC. Therefore, if our results indeed reflect a causal link between AGEs and CAC, this might mean that cross-linking or intracellular glycation via MGO may stimulate the process of arterial calcification. The fact that we did find an association of pentosidine and THP with CAC in individuals with T1DM who were relatively young and had an early stage of CAC may indicate that increased levels of these AGEs are associated with early development of CAC. However, overall, our numbers of individuals with substantial calcification in the study are low, probably partially due to the relatively young age of the participants, which is why these analyses should be replicated in larger cohorts.

AGEs are able to induce LGI and ED [3,5,40], and markers of these processes are associated with coronary or carotid artery disease in T1DM [41,42]. We demonstrated that the association of pentosidine with CAC was not explained (i.e. mediated) by LGI or ED. Other studies that investigated the mediating effect of LGI and ED in the association of AGEs with CVD in T1DM [3,43] also found no mediating effect of either of these potential mechanisms. Therefore, other mechanisms besides LGI and ED might be involved in the association of AGEs with CAC. Recent publications show that AGEs-induced vascular calcification in rat vascular smooth muscle cells (VSMCs) is mediated by oxidative stress in vitro [44,45], and oxidative stress may thus provide an additional mechanism explaining the association of AGEs with CAC.

The association between pentosidine and CAC might be causal since experimental studies have demonstrated a direct link of AGEs with calcification [21]. In response to AGEs, aortic VSMCs differentiate into cells that exhibit an osteoblast-like phenotype characterized by the deposition of calcium into the extracellular matrix [19,20]. These findings support a direct role of AGEs in the stimulation of CAC. Furthermore, the AGE-RAGE axis has been associated with arterial calcification in animal studies [22,23]. However, in our study, we did not find an association between CML, a known ligand for RAGE, and CAC. Another pathway by which AGEs are thought to contribute to atherosclerosis is by the stimulation of apoptosis of endothelial progenitor cells (EPCs) and by the impairment of EPC functions [46,47]. Indeed, skin autofluorescence, an estimate of tissue AGE accumulation, has been negatively associated with circulating EPCs [48]. Furthermore, low levels of EPCs have been shown to be an independent determinant of carotid intima media thickness (cIMT) in young individuals with T1DM [49]. Interestingly, in individuals with compared to those without coronary atherosclerosis, it was found that a higher percentage of EPCs express the osteoblastic marker osteocalcin (OCN) [50], which has been shown to correlate with markers of bone formation [51]. Therefore, a particular subset of EPCs has been suggested to mediate abnormal vascular repair and vascular calcification [52].

### Limitations of our study

Our study had a cross-sectional design; therefore we cannot draw any conclusions about causality in the association of AGEs with T1DM and CAC. Because not all atherosclerotic plaques contain calcium, the CAC score does not take non-atherosclerotic plaques into account. Despite this caveat, it has been shown that CAC is highly associated with total coronary atherosclerotic plaque burden [53]. Additionally, individuals with diabetes are known to have a higher prevalence of medial calcification of the peripheral vessels. However, medial calcification of the coronary tree, not caused by atherosclerosis, is not very common in diabetes [54]. It therefore seems likely that the CAC score measured in our study indeed reflects intima calcification associated with atherosclerosis.

We cannot discard the possibility that the use of a single or a selection of markers representing LGI and ED, respectively, may have led to an underestimation of their mediating effects in the association of AGEs with CAC. However, hsCRP is one of the most studied and best validated markers and is thought to represent overall LGI. Furthermore, hsCRP is known to be associated with coronary heart disease [55]. sVCAM-1 and vWF are well known markers of ED [56].

## Conclusions

In conclusion, we found higher plasma levels of the AGEs pentosidine, CML and THP in individuals with compared to without T1DM, independent of age, sex, body mass index, waist-to-hip ratio, smoking, blood pressure, lipid profile and glomerular filtration rate. Pentosidine levels were higher in individuals with T1DM with a moderate to high compared to a low CAC score, independent of age, sex, body mass index, waist-to-hip ratio, smoking, blood pressure, lipid profile and glomerular filtration rate. The association of pentosidine with CAC was not explained (i.e. mediated) by LGI or ED. These results may indicate that AGEs are involved in the development of CAC in T1DM, but future studies are needed to fully elucidate the direction and potential causality of this relationship. If AGEs indeed play a role in the early development of coronary artery calcification in T1DM, they could be an early target for the prevention of CVD.

## Abbreviations

AGE: Advanced glycation end-product; ANCOVA: Analysis of covariance; BMI: Body mass index; CAC: Coronary artery calcification; CEL: Nϵ-(Carboxyethyl)lysine; CML: Nϵ-(Carboxymethyl)lysine; CT: Computed tomography; CVD: Cardiovascular disease; CVs: Coefficients of variation; DBP: Diastolic blood pressure; EBCT: Electron-beam computed tomography; ED: Endothelial dysfunction; EDTA: Ethylenediaminetetraacetic acid; eGFR: Estimated glomerular filtration rate; eGFR: Estimated GFR; ELISA: Enzyme-linked immunosorbent assay; HbA1c: Glycated hemoglobin; HDL: High-density lipoprotein; HPLC: High performance liquid chromatography; hsCRP: High-sensitivity C-reactive protein; LDL: Low-density lipoprotein; LGI: Low-grade inflammation; LYS: Lysine; MDRD: Modification of Diet in Renal Disease; MGO: Methylglyoxal; MS/MS: Tandem mass-spectrometry; RAGE: Receptor for AGEs; SBP: Systolic blood pressure; sVCAM-1: Soluble vascular cell adhesion molecule 1; sβ: Standardised β; T1DM: Type 1 diabetes mellitus; THP: Tetrahydropyrimidine; UPLC: Ultra performance liquid chromatography; VSMCs: Vascular smooth muscle cells; vWF: Von Willebrand factor.

## Competing interests

The authors declare that they have no competing interests.

## Authors’ contributions

MGAE, MTS and CGS participated in the conception and design, analysed and interpreted the data, drafted the manuscript and provided final approval of the version to be published. HMC carried out the coronary artery calcification study, participated in the conception and design, analysed and interpreted the data, revised the paper for important intellectual content and provided final approval of the version to be published. JLJMS analysed and interpreted the data, revised the paper for important intellectual content and provided final approval of the version to be published. CDAS participated in the conception and design, analysed and interpreted the data, revised the paper for important intellectual content and provided final approval of the version to be published. All authors read and approved the final manuscript.
